# Maternal factors associated with low birth weight in public hospitals of Mekelle city, Ethiopia: a case-control study

**DOI:** 10.1186/s13052-020-00890-9

**Published:** 2020-09-07

**Authors:** Sielu Alemayehu Desta, Ashenafi Damte, Tsehay Hailu

**Affiliations:** School of nursing, college of health science, Mekelle University, Mek’ele, Ethiopia

**Keywords:** Low birth weight, Maternal, Factors, Case control

## Abstract

**Background:**

Mothers in low socio-economic conditions frequently have low birth weight infants. Inaddition Physically demanding work during pregnancy also contributes to poor fetal growth. During gestation a woman needs balanced nutrition for a healthy outcome. Women with inadequate nutritional status at conception are at greater risk of aquiring disease; their health usually depends on the availability and consumption of balanced diet, and therefore they are unlikely to be able to resist with their high nutrient needs during pregnancy. Therefore, the main purpose of this study was to assess the maternal risk factors associated low birth weight in public hospitals of Mekelle city, Tigray North Ethiopia, 2017/2018.

**Methods:**

Un-matched case-control study design was conducted among women who delivered in public hospitals of Mekelle city. Data was collected using a structured questionnaire through interview, direct physical assessment and medical record review of mothers. Sample size was calculated by Epi-info version 7.0 to get a final sample size of 381(cases = 127 and controls = 254). SPSS version 20 was used for analysis. Bivariate and multivariate logistic regression analysis was used to determine the effect of the independent variables on birth weight. Presence of significant association was determined using OR with its 95%CI. A *P* value of less than 0.05 was considered to declare statistical significance. Table, graphs and texts were used to present the data.

**Result:**

Most of the mothers (70.1% cases and 43.7% controls) were housewives. This study showed that maternal age ≤ 20 years (AOR = 6.42(95% CI = (1.93–21.42)), ANC follow up (AOR = 3.73(95%CI (1.5–9.24)), History of medical illness (AOR = 14.56(95% CI (3.69–57.45), Iron folate intake (AOR = 21.56(95%CI (6.54–71.14)), Maternal height less than 150 cm (AOR = 9.27(95%CI 3.45–24.89)) and Pregnancy weight gain (AOR = 4.93(95%CI = 1.8–13.48) were significant predictors of low birth weight.

**Conclusion:**

The study suggests that inadequate ANC follow-up, preterm birth and history of chronic medical illness, maternal height, pregnancy weight gain, and Iron intake were. Were significant predictors of low birth weight. Health professionals should screen and consulate pregnant mothers who are at risk of having infants with LBW and ensure that women have access to essential health information on the causes of low birth weight.

## Background

World Health Organization (WHO) defined low birthweight as weight at birth of less than 2500 g regardless of gestational age. This cut-off point is based on observations that newborns weighing less than 2500 g. are 20 times more likely to die than heavier babies [1]. LBW can be further subcategorized as very low birth weight (VLBW), which is less than 1500 g. and extremely low birth weight (ELBW), which is less than 1000 g [2].

Low birthweight is caused by either due to preterm birth (born before 37 weeks of gestation) or the infant being small for gestational age (slow prenatal growth rate) or a combination of both [3]. The majority of LBW neonates in developing countries are due to small for gestational age whereas preterm birth is the most common cause of LBW in industrialized countries [4]. Small for gestational age can occur with unknown cause or it can be secondary to intrauterine growth restriction that is related to many possible factors, like congenital anomalies and Infections. In addition Problems with the placenta can prevent it from providing adequate oxygen and nutrients to the fetus [5].

In many cases, the exact causes of prematurity are unknown; but they may associated with high maternal blood pressure, acute infections, hard physical work, multiple births, stress, anxiety, and other psychological factors [4]. Multiple factors of LBW have been identified, which include genetics, early labor, multiple pregnancy, various maternal illnesses (i.e. pregnancy-induced hypertension, diabetes mellitus and infections), drug abuse (including tobacco and alcohol), maternal age, height nutritional factors (underweight, overweight and obesity) [1, 6].

Mothers in low socio-economic conditions frequently have low birth weight infants. Inaddition Physically demanding work during pregnancy also contributes to poor fetal growth [1, 7]. During gestation a woman needs balanced nutritional for a healthy birth outcome [8]. Women with inadequate nutritional status at conception are at greater risk of aquiring disease; their health usually depends on the availability and consumption of balanced diet, and therefore they are unlikely to be able to resist with their high nutrient needs during pregnancy [6].

Majority of low birth weight children have higher rates of subnormal growth, illnesses, and neurodevelopmental problems. These issues increase becuase the child’s birth weight decreases. Low birth weight infants are prone to have difficulties in cognition, attention, and neuro muscular functioning [9]. Worldwide More than 20 million infants are born annually out of them 15.5% are born with low birth weight (LBW), 95.6% of them are from developing countries. The level of LBW in developing countries is more than twenty fold than the level in developed countries [1]. About17 million infants are born with LBW in developing countries annually. Many of those infants who can survive were suffered with cognitive and neurological impairment In fact, a child born with low birth weight has, a greater risk of illness and premature death from cardiovascular disease, hypertension, and diabetes, in later life compared to others with adequate birth weights [4].

Majority of LBW births occur in low- and middle-income countries and especially in the most vulnerable populations. Regional estimates of LBW include 28% in south Asia, 13% in sub-Saharan Africa and 9% in Latin America but it is also a global concern, to some high-income countries (e.g. Spain, the United Kingdom of Great Britain and Northern Ireland and the United States of America [7].

According to the in depth analysis of Ethiopian demographic and health survey 2016 the prevalence of LBW in Ethiopia was about 29.1% [10]. and a study done in Tigray, Ethiopia showed that the prevalence of LBW in Rural and urban is about 9.9 and 6.3% respectively [11]. Many studies stated that there is great association between maternal health, nutrition, Socio Demographic, Obstetric and gynecological factors with the incidence of LBW [12, 13].

Child who have low birth weight children have immature immune function are also prone to have increased risk of disease, lower IQ and cognitive disabilities which could affect their performance in school, job opportunities as adults and may develop chronic illness like diabetes and coronary heart disease in adult hood [6]. Low Birth Weight of is also known to cause cerebral palsy more frequent hospitalization for all illness, more hearing and visual disability more behavioral disorders [7].

Even though some studies have been tried to assess determinants of LBW in the country no published studies has been yet conducted on identifying maternal risk factors with low birth weight in this study setting. And most of the efforts that have been made before to identify the predictors were outdated that made it difficult to know the current situation. So this study is aimed at identifying maternal risk factors with low birth weight by using comparative group that will help as a base for other researchers, health care providers, and policy makers for further designing of strategic plan and intervening accordingly.

## Methods

### Study area and study period

The study was conducted in Mekelle city, Tigray, Ethiopia. Mekelle is a capital city of Tigray regional state and one of the administrative towns. The city is located in the northern part of Ethiopia with a distance of 783 km from Addis Ababa, the capital city of Ethiopia. Its astronomical location is 13°32″North latitude and 39°28′ East longitude. The city has total population of 586,897 according 2015 EFY. In the city are about 12 public health centers and 4 public hospitals providing promotive, preventive, curative, and rehabilitative services. The health institutions in the city give maternal and child health services. The study was carried out from February to March 2018.

### Study design

Institutional based unmatched case-control study design was conducted among women who delivered in public hospitals of Mekelle city from November 2017 to June2018.

### Source population

All mothers who delivered at public hospitals of Mekelle, Tigray, Ethiopia during the study period.

### Study population

#### Cases

Mothers who delivered low birth weight neonate (< 2500 g) at public hospitals of Mekelle City, Tigray, Ethiopia from February to March 2018.

#### Controls

Mothers who delivered normal birth weight neonates (2500–4000 g.) in public hospitals of Mekelle City, Tigray, Ethiopia from February to March 2018.

### Inclusion criteria

- For cases; low birth weight child (< 2500 g) singleton live births
- For controls; Normal birth weight (2500-4000 g) singleton live births

### Exclusion criteria

For all cases and controls; Newborns with congenital anomalies and critically ill mothers were excluded from the study.

### Sample size determination

Double population proportion formula using Epi-info version 7.0 statistical package was used considering maternal height (≤ 150 cm) as main exposure variable, percent of exposure for controls 6.2% (taken from a study conducted in Bale) [14]. And an assumptions of 95% CI, 80% power, case to control ratio of 1:2 and 2.8 odds ratio was used to get a total sample size of 345. Adding 10% non-respondent rate the final sample size was *n* = 381(cases = 127, controls = 254).

### Sampling procedure

**Fig. 1:**
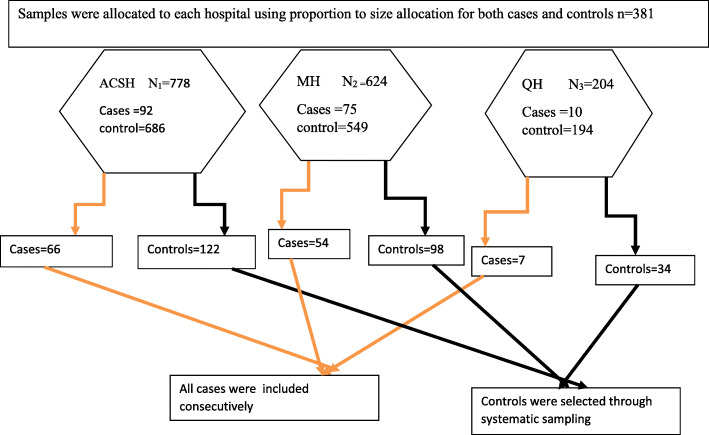
Schematic presentation of the sampling procedure for a study conducted on maternal risk factors associated with LBW

All public hospitals in Mekelle city (Ayder Comprehensive Specialized Hospital, Quiha and Mekelle general hospitals) were included on the study. Both cases and controls were proportionally allocated to each hospitals by taking their average flow of deliveries for the last 3 month as a baseline. Averagely in 3 months there were about 1606 neonates delivered in those three public hospitals of Mekelle city. Among these 778 were in ACSH, 624 in Mekelle and 204 in Quiha hospital. All cases in each hospital were included consecutively until the required sample siz were obtained and controls were recruited using systematic random sampling by selecting the participants every 3rd interval [Fig. 1].

### Study variables

#### Dependent variable

Low Birth weight.

#### Independent variables

**Maternal Socio-demographic factor** (Maternal age, Residence, Educational level and Maternal occupation).

**Maternal nutritional factors** (Gestational weight gain, Height, Weight, and Iron and folic acid supplementation).

**Maternal obstetric and health –related factors** (Birth interval, Gestational age, Gestational and chronic medical illness, History of abortion and Number ofANC follow up).

**Maternal behavioral factors (**Drinking alcohol and Cigarette smoking).

### Operational definitions

#### Normal birth weight range

Newborns who have birth weight (2500 g. -4000 g.)

#### Case

Mothers who delivered low birth weight neonate (< 2500 g.)

#### Control

Mothers who delivered normal birth weight neonates (2500–4000 g.)

#### Birth interval

Birth interval is defined as the length of time between two successive live births.

### Data collection tools

Data were collected using a structured English version questionnaire which was adapted from different literatures. The socio- demographic and behavioral maternal factors were collected through interview. Maternal anthropometric measurements like Height was computed through physical assessment and ANC, gestational age and any relevant medical illness were extracted through reviewing of mothers’ medical record for both cases and controls within the first 6 h of delivery. Data collectors were interviewed to all mothers for whom who have singleton live births all over the data collection period at the selected hospitals for both controls and cases. Birth weight of every child was measured using balanced seca scale and the scale was rounded to the nearest 50 mg..

### Data quality control and management

Pretest was conducted in Wukro Hospital on 5% (in 7 cases and 14 controls) of study participants which were not included in the study prior to the actual data collection period to test the clarity, consistency and completeness of the questioner. Six data collectors (BSc. midwives) two for each hospital and one supervisor (BSc. midwifery) were trained for 1 day on how to collect, interview and the overall objectives of the study by principal investigator. English version Questioner were changed in to local language (Tigrigna) then translated back in to English for analysis. Weighing scales were checked and adjusted at zero level for the validity of the measurement. Data were managed by using appropriate data entry in to SPSS version 20 software package and it was cleaned before analysis.

### Data analysis

Affter the data were codded and cleaned it was entered to SPSS version 20 for analysis. Descriptive statistics such as mean (+SD) were calculated to compare group variables.. In the Binary logistic regression model bivariate analysis was run to include variables as a candidate in the multivariate logistic regression at *p* value of ≤0.2. A multivariate logistic regression was used to determine the effect of the independent variables on birth weight and to control possible confounders. In order to test the significance level and association of variables at 95% confidence interval (CI), adjusted odds ratio and *p*-value ≤0.05 were used. Tables, graphs and texts were used to present the data.

### Ethical consideration

Ethical clearance was obtained from the Institutional Ethical Review Board of Mekelle University College of Health Sciences and support letter was given fromTigray regional heath bureau to the selected hospitals letting permission. As long as reviewing mothers card and assessing mothers immediately after delivery needs verbal informed consent and confidentiality was preserved by apprising data collectors to use codes instead of writing names of the respondents and assuring the consent of respondents before data collection inorder to maintain permission of the participants. The informed consent was also applied for the newbons and and young mothers. The verbal consent was obtained from a parent on behalf of the participants under the age of 16.

## Results

**Fig. 2:**
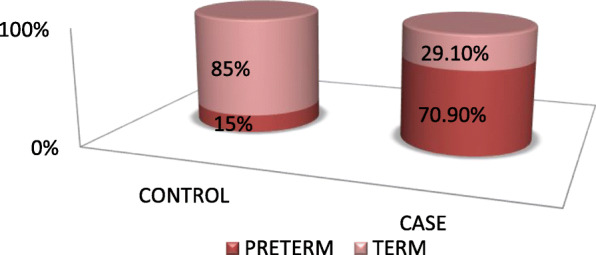
Distribution of low birth weight among preterm and post term babies in Mekelle Tigray Ethiopia 2018

A total of 381 (127 cases and 254 controls) newborns were included in this study making a response rate of 100%. The mean birth weight was about 1966.6 g (S.D ± 359.58 g) for cases and 3125.gm (S.D ± 384.86) for the controls. About 70.9 and 29.1% of the cases were Preterm and term respectively [Fig. 2].

### Maternal socio- demographic characteristics

Fifty three (41.7%) of the mothers of the cases and 180(70.9%) controls were found in the age group 21–35 years. The minimum and maximum age of both mothers of both the cases and controls were 15 years and 45 years respectively. About 85% percent of mothers of the cases and 81.5% of mothers of the controls were orthodox. Larger proportions of cases (70.1%) and controls (43.7%) were housewives. Fifty seven percent (57.5%) of cases and (69.3%) of the controls reside in urban area. [Table 1].

**Table 1:** Distribution of mothers by socio demographic characteristics in Mekelle, city, Tigray

Variables	Cases (127)	Controls (254)	Total (*n* = 381)
	Frequency (%)	Frequency (%)	Frequency (%)
**Age**			
≤ 20	46 (36.2%)	16 (6.3%)	62 (16.3%)
21–35	53 (41.7%)	180 (70.9%)	233 (61.1%)
≥ 36	28 (22%)	58 (22.8%)	86 (22.6%)
**Religion**			
Orthodox	117 (92.1%)	207 (81.5%)	324 (85%)
Muslim	6 (4.7%)	29 (11.4%)	35 (9.2%)
Others	4 (3.1%)	18 (7.1%)	22 (5.8%)
**Residence**			
Rural	54 (42.5%)	78 (30.7%)	132 (34.6%)
Urban	73 (57.5%)	176 (69.3%)	249 (65.3)
**Ethnicity**			
Tigray	125 (98.4)	225 (88.6%)	350 (91.9%)
Amhara	2 (1.6%)	15 (5.9%)	17 (4.5%)
Other	0 (0%)0	14 (5.5%)	14 (3.7%)
**Marital status**			
Single	29 (22.8%)	61 (24.0%)	90 (23.6%)
Married	92 (72.4%)	177 (69.7%)	269 (70.6%)
Other	6 (4.7%)	16 (6.3%)	22 (5.8%)
**Educational status**			
Not read and write	26(2o.5%)	40 (15.7%)	66 (17.3%)
primary education	22 (17.3%)	18 (7.1%	40 (10.5%)
Read and write	7 (5.5%)	16 (6.3%)	23 (6%)
Secondary education	55 (43.3)	110 (43.3%)	165 (40.3%)
collage/university	17 (13.4%)	70 (27.6%)	87 (22,8%)
**Occupation of the mother**			
House wife	89 (70.1%)	111 (43.7%)	200 (52.4%)
Merchant	14 (11.0%)	33 (13.0%)	47 (12.3%)
Governmental employed	24 (18.9%)	110 (43.3%))	134 (35.2%)

### Maternal obstetric characteristics

Almost half of the mothers of the cases (46.5%) and controls (42.5%) were primi-gravida. Most mothers of the controls (86.2%) and cases (81.9%) had no history of abortion. About 73(57.5%) of the cases had 1–2 ANC visits while 169(66.5%) of the controls had 3–4 ANC visits during their pregnancy. Fourty prcent of the cases and 16.1% the controls had pregnancy related complication in which PIH accounts 20.5 and 6.3% of the complications for cases and controls respectively. (Table 2).

**Table 2:** Distribution of mothers by obstetric and health related- factors in Mekelle cityTigray

Obstetric and health related factors	Cases (*n* = 127	Controls (*n* = 254	Total (*n* = 381)
	Frequency (%)	Frequency (%)	Frequency (%)
Number of previous births			
Primi	59 (46.5%)	108 (42.5%)	167 (43.8%)
1	15 (11.8%)	32 (12.6%)	47 (12.4%)
2–3	53 (41.7%)	114 (44.9%)	167 (43.8)
Pregnancy interval (in months)			
Primi	59 (46.5%)	95 (37.4%)	154 (40.4%)
6–24	28 (22%)	57 (22.4%)	85 (22.3%)
25–36	24 (18.9%)	63 (24.8%)	87 (22.8%)
37–48	14 (11.0%)	29 (11.4%)	43 (11.3
> = 48	2 (1.6%)	10 (3.9%)	12 (3.2%)
History of abortion			
No	104 (81.9%)	219 (86.2%)	323 (84.8%)
Yes	23 (18.1%)	35 (13.8%)	58 (15.2%)
ANC visit			
No	18 (14.2%)	26 (10.2%)	44 (11.5%)
Yes	109 (85.8%)	228 (89.8%	337 (88.5%)
No of ANC visit			
NO	18 (14.2%)	26 (10.2%	44 (11.5%)
1–2	73 (57.5%)	59 (23.2%)	132 (34.6%)
3–4	36 (28.3%)	169 (66.5%)	112 (53.9%)
pregnancy related complication during the current pregnancy			
No	76 (59.8%)	213 (83.8%)	289 (75.8%)
Yes	51 (40.2%)	41 (16.1%)	92 (24%)
Type of Pregnancy related complication			
No	76 (59.8%)	213 (83.8%)	289 (75.8%)
APH	11 (8.7%)	10 (3.9%)	21 (5.5%)
PROM	10 (7.9%)	4 (1.6%)	14 (3.7%)
PIH	26 (20.5%)	16 (6.3%)	42 (11%)
DM	4 (3.1%)	11 (4.3%)	15 (3.9%)
History of any chronic medical illness pregnancy			
No	95 (74.8%)	244 (96.1%)	339 (88.8%)
Yes	32 (25.2%)	10 (3.9%)	42 (11.8%)
Hx of malaria infection during the current pregnancy			
No	111 (87.4%)	228 (89.8%)	339 (88.8%)
Yes	16 (12.6%)	26 (10.2%)	42 (11.8%)

### Maternal nutritional and behavioral characteristics

From the total participants 88.2% of cases and 92.1% of the controls had taken iron folate during their pregnancy period but they differ in number of iron folate they took i.e. 9.4% of the cases and 44.1% of the controls had taken greater than 90 tablets during their pregnancy. 11.8% of the controls and 42.5% of the cases were with the height of < 150 cm. In addition 35.4% of the controls and 4.7% of the cases have gestational weight gain of > 12 kg (Table 3). Almost all participants from both groups have no Hx of Cigarette smoking (Table 3).

**Table 3:** Distribution of mothers by nutritional and behavioral factors in Mekelle city Tigray

Maternal nutritional factors	Classification of Participants		
	Case	Control	
	Frequency (%)	Frequency (%)	Total (%)
Height of mother			
< 150 cm	54 (42.5%)	30 (11.8%)	84 (22%)
≥ 150 cm	73 (57.5%)	2 24 (88.2%)	297 (78%)
weight gain during pregnancy			
< 12 kg	121 (95.3%)	164 (64.6%)	285 (74.8%)
≥12 kg	6 (4.7%)	90 (35.4%)	96 (25.2%)
Have you ever taken iron folate			
No	15 (11.8%)	20 (7.9%)	35 (9.2%)
Yes	112 (88.2%)	234 (92.1%)	246 (90.8%)
How many tablets did you take			
No	15 (11.8%)	20 (7.9%)	35 (9.2%)
< 60	75 (59.1%)	45 (17.7%)	120 (31.5%)
60–90	25 (19.7%)	77 (30.3%)	102 (26.8%)
> 90	12 (9.4%)	112 (44.1%)	124 (32.5%)
Have you ever drink alcohol			
No	49 (38.5%)	112 (44%)	161 (42.2%)
Yes	78 (61.4%)	142 (55.9%)	220 (57.7%)
How often do you have drink			
No	49 (38.5%)	112 (44%)	161 (42.2%)
once or twice	11 (9%)	33 (13%)	44 (11.5%)
Monthly	45 (35%)	79 (31%)	124 (32.5%)
Weekly	22 (17%)	30 (12%)	52 (13.6%)

### Factors associated with low birth weight

Bivariate logistic regression analysis was performed between maternal associated factors and low birth weight. The finding revealed that maternal age, residence, maternal educational status, maternal occupation, number of ANC follow up, pregnancy related complication, type of pregnancy related complication, history of chronic medical illness, number of iron tablet,height of mother, pregnancy weight gain and gestational age were statistically significant with low birth weight in the bivariate model.

But after adjusting the possible confounders only maternal age, ANC follow up, history of medical illness, Iron intake, maternal height and maternal weight were found to significantly associated with low birth weight. Maternal age ≤ 20 years were six times more likely to deliver low birth weight than mothers with age ≥ 36 (AOR =; 6.42(95% CI (1.93–21.42), Those mothers with 1–2 ANC follow up were three times more likely to have low birth weight baby than mothers having 3–4 ANC follow up (AOR = 3.73(95%CI (1.5–9.24)). Mothers who had history of medical illness were at risk to give low birth weight baby as compared to mothers with no history of chronic medical illness (AOR = 14.56(95% CI (3.69–57.45));

Mothers who take iron folate < 60 were twenty one times more likely to deliver low birth weight babies than those who take > 90 tablets (AOR = 21.56(6.54–71.14)). Mothers with height < 150 cm were nine times more likely to deliver low birth weight baby than their counter part (> = 150 cm) with (AOR = 9.27(95%CI 3.45–24.89) and mothers who gain weight < 12 kg during pregnancy were at higher risk than those who gain > = 12 kg (AOR = 4.93(95%CI 1.8–13.48)).preterm babies were fourteen times more likely to be low birth weight than term babies (AOR = 14.28((95%CI = 5.75–35.47). (Table 4).

**Table 4:** Maternal associated factors of low birth weight (LBW) of Mekelle city, Tigray, Ethiopia (*n* = 381)

Factors	Cases	Controls	COR (95% CI)	AOR (95%CI)
Number of ANC	Frequency (%)	Frequency (%)		
NO	18 (14.2%)	26 (10.2%)	3.25 (1.61–6.55)	3.22(.297–34.8)
1–2	73 (57.5%)	59 (23.2%)	5.81 (3.53–9.55)	3.77 (1.53–9.32)
3–4	36 (28.3%)	169 (66.5%)	1	1
Hx of chronic medical illness				
NO	95 (74.8%)	244 (96.1%)	1	1
Yes	32 (25.2%)	10 (3.9%)	8.22 (3.89–17.37)	14.56 (3.69–57.45)
How many tablet				
N0	15 (11.8%)	20 (7.9%)	7 (2.86–17.15)	5.02(.37–68.58)
< 60	75 (59.1%)	45 (17.7%)	15.56 (7.72–31.35)	21.56 (6.54–71.14)
60–90	25 (19.7%)	77 (30.3%)	3.03 (1.44–6.4)	2.14(.63–7.3)
> =90	12 (9.4%)	112 (44.1%)	1	1
Height of mother				
< 150 cm	54 (42.5%)	30 (11.8%)	5.52 (3.29–9.28)	9.27 (3.45–24.89)
> =150 cm	73 (57.5%)	2 24 (88.2%)	1	1
Wt. gain during pregnancy				
< 12 kg	121 (95.3%)	164 (64.6%)	11.08 (4.687–26.1)	4.93 (1.8–13.48)
> =12 kg	6 (4.7%)	90 (35.4%)	1	1
Gestational age				
< 37wks	90 (70.9%)	38 (15%)	14.1 (6.082–32.7)	14.28 (5.75–35.47)
> =37	37 (29.1%)	216 (85%)	1	1
Age Category				
< 20	46 (36.2%)	16 (6.3%)	5.96 (2.88–12.31)	6.421 (1.93–21.42)
21–35	53 (41.7%)	180 (70.9%)	.61(.35–1.05)	0.867(.32–2.39)
> =36	28 (22%)	58 (22.8%)	1	1

## Discussion

LBW is still a significant cause of morbidity and mortality among neonates. This study has tried to assess determinants of LBW among mothers who gave birth at public hospitals of Mekelle city. Results of this study found that maternal age was a significant predictor of low birth weight. This result is in line with studies conducted in Brazil, India, and Bale [12, 14, 15]. This similarity may be due the sharing of similar techniques used and life style of the participants. But this result is different in contrast with a study done in West Iran [13]. This descrepancy might be due the difference in socio demographic characteristics of study participants and difference in the techniques and mothedes of the study.

This study also revealed that mothers who did not follow ANC were three times more likely to have low birth weight baby than mothers who have 3–4 ANC follow up. This finding is in agreement with a study done in Eastern Nepal, Addis Ababa and Axum [16–18]. This agreement may be due to the fact that continuous ANC visit can help to ensure continuous interventions and assessment of those mothers at risk and allow time to intervene activities like nutritional education, pregnancy related complications and other adverse outcome of pregnancy.

In this study mothers who took < 60 iron folate tab were twenty one times more likely to deliver low birth weight babies than those who take > 90 tablets. This result is similar with studies conducted in Eastern Nepal and Tigray [17, 19]. which shows significant association of iron folate with the weight of new born. This can be cuased because the growing fetus shares not only iron but also other nutrient from mother.so that mothers needs complementary iron to compensate for the intrauterine development of the fetus [20].

This study revealed that Maternal height < 150 cm was found as significant predictor of LBW this is congruent with study done in Bale and India [14, 15]. The risk estimates for having an infant with LBW was significantly elevated for women with short stature (height < 150 cm). This might be because of height of a mother is an outcome of several factors including nutrition during her childhood and adolescence and Slower fetal growth can occur due to short maternal stature.

In this study preterm baby were fourteen times more likely to be low birth weight than term babies this is similar with study done in Addis Ababa and Axum [16, 18]. This is due to the known fact that as gestational age of the fetus decreases from the standard gestational age (37 week) the body weight of the fetus falls dramatically due to prematurity.

Pregnancy weight gain < 12 kg was another significant predictor of LBW .this is in line with study done in India [15]. This might be due to insignificant weight gain during pregnancy is a marker for marginal tissue nutrient and a predctor of protein-energy malnutrition, which may affect fetal growth [21].

### Limitation of the study

- Since the design of this study is institution based case control study, it may have limited the generalizability of the findings to the community.
- Also, this study selectively addressed certain factors of low birth weight while various factors are found to cause the diseases.

## Conclusion

The study suggests that inadequate ANC follow-up, history of chronic medical illness, maternal height, pregnancy weight gain, and Iron intake were significant predictors of low birth weight. Health professionals should screen and counsel pregnant mothers who are at risk of having infants with LBW and ensure that women have access to essential health information on the causes of low birth weight. Mothers should receive problem (low birth weight) specific counseling by skilled health personnel with emphasis given to those with chronic medical illnesses prevention of preterm Nutritional education to improve the weight gain during pregnancy, and prevention and proper management of chronic medical illness.

## Supplementary information

**Additional file 1.** Questionnaie Tsehay. Questionnaire. The data contains the questionnaire for collecting information on maternal risk factors associated with low birth weight. The questionnaire has four sections. The first section is concerned with datas related to newborn characteristics, the second section is concerned with socio demographic characteristics while the second and third sections are concerned with Obstetric and gynecological history and maternal nutritional factors respectively.

## Data Availability

The datasets used and/or analyzed during the current study are available from the corresponding author on reasonable request.

## Abbreviations

ACSH: Ayder Comprehensive Specialized Hospital
ANC: Antenatal Care
AOR: Adjusted Odd Ratio
CI: Confidence Interval
COR: Crude Odd Ratio
EDHS: Ethiopian Demographic and Health Survey
IUGR: Intrauterine Growth Retardation
LBW: Low Birth Weight
MUAC: Middle Upper Arm Circumstance
SGA: Small for gestational age
SPSS: Statistical Package for Social Sciences
WHO: World Health Organization
