# Supplementary material for: Maternal factors associated with low birth weight in public hospitals of Mekelle city, Ethiopia: a case-control study
Source: Ital J Pediatr. 2020 Sep 7;46:124. doi: 10.1186/s13052-020-00890-9 (PMC7487657; doi:10.1186/s13052-020-00890-9)
Supplement: Supplementary file 1 — Additional file 1. Questionnaie Tsehay. Questionnaire. The data contains the questionnaire for collecting information on maternal risk factors associated with low birth weight. The questionnaire has four sections. The first section is concerned with datas related to newborn characteristics, the second section is concerned with socio demographic characteristics while the second and third sections are concerned with Obstetric and gynecological history and maternal nutritional factors respectively. [file 13052_2020_890_MOESM1_ESM.docx]

**Questionnaire to maternal factors associated with low birth weight in public hospitals of Mekelle city**

**Information sheet**

TITLE: Maternal riskfactors associated with low birth weight in public hospitals of Mekelle, Tigray, North Ethiopia: a case-control study.

Institution: Mekelle University, College of Health Sciences Department of Nursing

Name of sponsor: Mekelle University

Principal Investigator: Sielu Alemayehu (BSc.)

Mobile: +251 920437801

E-mail: sielu.alemayehu@mu.edu.et

**Background Information**: Low birth weight is defined as birth weight less than 2,500 grams. More than 20 million infants are born each year weighing less than 2500 gm. accounting for 17% of all births in the developing world, Birth weight plays an important role in infant mortality morbidity, development, and future health of the child. Weight at birth is directly influenced by general level ofSocio demographic, nutritional and health status of the mother.

Address of the principal investigator: Tsehay Hailu Phone Number: +251920437801

E-mail: [tsehay123hailu@gmail.com](mailto:tsehay123hailu@gmail.com)

**Consent form**

Mekelle University College of Health Sciences School of Nursing

Questionnaire for Assessment of Maternal risk Factors Associated with Low Birth Weight in Public Hospitals of Mekelle City, Tigray, North Ethiopia2017/2018.

INTRODUCTION

Greetings! My name is ______________________________. I am here representing a team to conduct data collection among mothers who gave birth to a live baby (either low birth weight or normal birth weight) about the maternal risk factors associated with low birth weight. Low birth weight is defined as birth weight less than 2,500 grams.

The main Objective of the study is to identify the maternal risk factors associated with low birth weight in Mekelle city. During the study your response will be kept confidentially, there is no name identified and there will be no any visible risk with you and your newborn baby being you are participant except some time consuming (around 30 minutes). There is no payment for your participation but we greatly thank for your participation and you have the right to refuse from participation at any time.

Are you voluntary to participate?

1. Yes I have understand the above information and I am volunteer to participate in a study conducted on low birth weight and maternal risk factors associated in Mekelle city Tigray region ,Ethiopia from November 2017toJune2018.

2 .No, if you are not voluntary to participate please stop here.

If you are voluntary to participate in the study; we kindly request you to provide your truthful response for the interview. Thank you for your participation.

**English version questionnaire**

This is a data collection format to assess the maternal risk factors associated with low birth weight in public hospitals of Mekelle city Tigray, North Ethiopia**.**

Name of Data collector: ------------- Date: --------------Qualification: ----------------

Data Collector agreement

“I certify that I have filled the questionnaire in accordance with the training that is given to meand instructions stated in it. I have confirmed that the information in it is correct.”

Signature-------------------------------------- Date--------------------------------

Name of the health facility: ----------------------------------

Checked by supervisor for completeness; Supervisors Name: -----------------------Signature—

| Note the inclusion criteria from clinical records:   Is the child born single 1: YES 2: NO   Is the child free of any visible major birth defect 1: YES 2: NO  If ―Noto any of the inclusion criteria, stop the collection of data.  If the New born weight < 2500 gm. make write in the box near to case and if new born  Weight>=2500 gm. make write in the box near to control.  1. Case  2. Control | | | | | | | | | | |
| --- | --- | --- | --- | --- | --- | --- | --- | --- | --- | --- |
| Health institution/facility type/ Codes | | | | | | | | | | |
|  | Section I New born characteristics | | | | | | | |  | |
| Code | Question | Response | | | | | | | Skip | |
| 101 | Birth weight baby in grams? | Birth weight in grams__________ | | | | | | |  | |
| 102 | What is sex of new born? | 1 Male  2 female | | | | | | |  |  |
| 103 | What is the gestational age at delivery,  in weeks? | Gestational age at delivery in weeks:  ________ | | | | | | |  |  |
|  | | | | | | | | | | |
| SECTION II Maternal Socio-demographic factors | | | | | | | | | | |
| Code | Question | | Response | | | | Skip | | | |
| 201 | Age of the mother at delivery | | ---------year. | | | |  | | | |
| 202 | Religion | | 1 Orthodox.  2 Muslim  3. Protestant  4. Others | | | |  | | | |
| 203 | Ethnicity | | 1. Tigre  2. Amhara  3. Afar  4. Other | | | |  | | | |
| 204 | Maternal Educational status | | 1. Not read and write  2. Read and write  3. Primary education  4. Secondary education and above | | | |  | | | |
| 205 | Where are you living? | | 1. Rural  2. Urban | | | |  | | | |
| 206 | Marital status | | 3. Single  4. Married  5. Divorced  6. Widowed | | | |  | | | |
| 207 | What is your occupation? | | 1 House wife  2. Merchant  3 Governmental employed  4 If other specify---------------- | | | |  | | | |
| Section III Maternal Obstetric and health- related factors | | | | | | | | | | |
| Code | Question | | | | | Response | | Skip | | |
| 301 | Number of previous births (include term  and premature deliveries) | | | | | 1.1  2.2-3  3.4-54.>=6 | | **Skip** If sheis primi to302 | | |
| 302 | What is the pregnancy interval of the previous birth? (months) | | | | | ______________ | | If she is primi  Skipto303 | | |
| 303 | Have you ever had abortion | | | | | 1 yes 2 No | | If no skip to 305 | | |
| 304 | How many abortion did you have | | | | | Number of abortions___ | |  | | |
| 305 | Did you have ANC visits for the current  Pregnancy? | | | | | 1. Yes 2. No | | If no skip to 307 | | |
| 306 | Number of ANC visits attended | | | | | ________________ | |  | | |
| 307 | Did you have pregnancy related complication | | | | | 1. Yes2. No | | If no skip to309 | | |
| 308 | What type of Pregnancy related complication? | | | | | 1. APH  2. PROM  3. PIH  4. DM 5.Other | |  | | |
| 309 | Did you have history of any chronic medical illness pregnancy? | | | | | 1 yes 2 no | | If no skip to311 | | |
| 310 | What type of illness? | | | | | 1 Hypertension  2 DM  3 Anemia  4 TB 5 If Other specify | |  | | |
| 311 | Did you have malaria infection during the  current pregnancy? | | | | | 1. Yes 2. No | |  | | |
|  | | | | | | | | | | |
| Section IV; Maternal Behavioral factors | | | | | | | | | |  |
| Code | Question | | | | Response | | | | Skip |  |
| 401 | Have you ever drink alcohol containing during the current pregnancy ? | | | | 1. Yes  2. No | | | | If no skip to403 |  |
| 402 | If yes How often do you have a drink alcohol containing during the current pregnancy | | | | 1.once or twice  2. monthly  3.weekly  4.daily | | | |  |  |
| 403 | Have you ever used tobacco during the current pregnancy? | | | | 1. Yes  2. No | | | | If no skip to 501 |  |
| 404 | How often do you used tobacco during the current pregnancy | | | | 1.Once or twice  2. monthly  3.weekly  4.daily | | | |  |  |
|  | Section; Maternal Nutritional factors | | | | | | | | |  |
| Code | Question | | | Response | | | | | Skip |  |
| 501 | Have you ever had taken Iron/folate during the current pregnancy? | | | 1. Yes  2. No | | | | | If no skip to 503 |  |
| 502 | If yes how many tablet have you ever taken? | | | 1. <60 tablets  2. 60-90 tablets  3. >90 tablets | | | | |  |  |
| 503 | Have you ever weight in the current first trimester pregnancy | | | 1. Yes  2. No | | | | |  |  |
| 504 | If yes what was your weight in the current first trimester pregnancy | | | Weight of mothers: __________ | | | | |  |  |
| 505 | Have you ever weight in the last third trimester? | | | 1. Yes  2. No | | | | |  |  |
| 507 | Height | | | _____________  _______________ | | | | |  |  |
| 508 | pregnancy weight gain | | | _______________ | | | | |  |  |

**11.4 Tigrigna version questionnaire**

ትግርኛ ሕታም መሕትት

ቅድመ መሕትት መፅናዕቲ. መብርሂ

መቐለ ዩኒቨርሲቲ ኮሌጅ ጥዕና ሳይንስ ክፍሊትምህርቲ ነርሲንግ

መፅናዕቲ: ኩነታትኣዶ፣ትሑትክብደትዘለዎዕሸልኣብምዉላድዘለዎፅዕንቶ

ስዒቡዘሎሓባሪፅሑፍንሕድሕድተሳተፌቲዝንበብእዩ፡፡

ደሓንዶሓዲርኪ/ዉዒልኪ፣ኣነ___________________________________________ዝተበሃልኩ እዚ ዝካየድ መፅናእቲ ኣመልኪቱ መረዲእታ ንምእካብ ካብመቐለ ዩንቨርስቲ፣ኮሌጅጥዕናክፍሊ ትምህርቲ ነርሲንግ ናይ ዝመፁ ጉጅለ መፅናዕቲ ሓደኣባል እየ፡፡ዕላማእዚመፅናዕቲኣብ 2010 ዓ/ም ኣብመቐለ ዝርከባ መንግስታዊ ሆስፒታላትዝወልዳ ኣዴታትኩነታትኣብትሑት ክብደትዘለዎዕሸልምዉላድ ዘለዎፅዕንቶ ንምፅናዕእዩ፡፡ንሰን ዝሓረያ፣እዚሓባሪ ፅሑፍድሕሪምንባበይ ትሕዝትኡ ተረዲአንን ምስታፍ እንተተሰማሚዐን እዩ፡፡ርእሲመፅናዕቲ፡ኣብ መቐለ ዝርከባመንግስታዊ ሆስፒታላትኣብ 2010 ዓ/ም ግሊጋልት ተጠቀምቲ ካብ ዝኾና ኣድታት ምስ ኣዶ ተዛማዴነት ዘለዎም ረቛሕታት፣ትሑት ክብደትዘለዎ ሕንጦ ኣብ ምዉላድ ዘለዎም ፅልዋ፡፡ኣብዚ መፅናዕቲ ብምስታፈን፤ቃለመሕትት መልሲ እንትህባና ግዜአን ናዓና ካብ ምሃብ ዝሓለፈ ዝበፅሐን ሓደጋ ኮነ ዘረኽበኦ ረብሓ ወይ ክፈሊት የለን፡፡ዝህባና ምላሽ ምሽጥራዉነቱ ዝተሓለወ፣ስመንይኹንዝህበኦ መረዲእታ ካብፌቃደ ንወፃኢ ናብ ሳሌሳይ ወገን ኣሕሊፍና ዘይንህብምኾና ነረጋግፅ፡፡ተሳተፎኽነ ብሙሉእ ፌቃደኝነተን ዝተመስረተ ኮይኑ ኣብ ከይዲ ቃለ መሕተት ማንኛዉም ሕቶን ከም ኣይግደዳን፡፡ነዚ መፅናዕቲ ኣመልኪቱ ሕቶተሃልይወን ነታዋናመፅንዒት ክረኽባይኽእላ፡፡

ሽምንኣዴራሻዋናመፅንዒት፡ፀሃይሃይሉ

ስልኪቐፅሪ:0920437801

ኢ-ሜይል tsehay123hailu@gmail.com

ንምስታፍተስማዕሚዐንዶ? እወ______________ ቐፅል

ኣይፋል__________ የቀንየለይ፡፡

ቅጥዒስምምዕነትቅድመመፅናዕቲሕቶንመልስን

ኣነ፣ንዚመፅናዕቲዝ

ተሓረኹተሳታፊትእቲናይሃበሬታፅሑፍብግቡእኣንቢበዓላማ፣ረብሓንመፅናዕቲከምኡዉንካባይትፅቢትዝግበርነገርንብምስታፈይምንምዓይነትሽግርከምዘይበፅሐኒተረዲአኣለኹ፡፡እዚመፅናእቲኣመልኪቱዝህቦመልሲስመይይኹንዝህቦመረዲእታካብፍቓደይወፃኢናብሳሌሳይወገንዘይመሓላለፍምዃኑተረዲአኣለኹ፡፡ኣብዚመፅናእቲንዘይምስታፋኮነኣብሞንጎእቲመፅናእቲኣብዝኾነእዋን

ንምቁራፅመሰለይሕሉዉምዃኑእዉንተረዲአኣለኹ፡፡

ፉርማተሳታፊይ _______________________

መለለይቑፅሪመሕትት __________

ሽምሓታቲ _____________ፉርማ _______ዕለት _______

ሽምመተሓባበሪ ____________ፉርማ _______ዕለት _______

**ትግርኛ መሕትት**

**መቐለ ዩኒቨርሲቲ ኮሌጅ ጥዕና ሳይንስ ክፍሊ ትምህርቲ ነርሲንግ**

**ኣብ ከተማ መቐለ ኣብ ዝርከባ መንግስታዊ ሆስፒታላት ኣብ2010 ዓም ዝወልዳ ኣዴታት ኩነታት ኣብ ትሑት ክብደት ዘለዎ ዕሸል ምዉላድ ዘለዎ ፅዕንቶ ንምፅናዕ ዝተዳለወ መሕትት::**

| ስዒቦም ኣብ ዘለዉ ረቛሕታት ተመስሪትካ ኩነታት ምክታት ወይ ዘይምክታት ኣብ መፅናዕቲ ዝሳተፈ(ዋና ተፅናዕትን፣ መነፃፅሪ ተፅናዕትን) ወስን፡፡   - ዕሸል እንትዉለድ ነፅሊ ድዩ ነይሩ 1:እወ 2:ኣይፋሉን - ዕሸል እንትዉለድ ካብ ዝኾነ ዓይነት ኣካሊዊ ጉዴኣት ነፃ ድዩ ነይሩ 1:እወ 2: ኣይፊሉን   መልሲ እዞም ሌዕል ኢልም ካብ ዝተዘርዘሩ ረቛሒታት ዋላ ሓደ እኳ ኣይፋሉን እንተኮይኑ ቓለ መሕትት ኣቛርፅ  ዕሸል እንትዉለድ ክብደቱ ትሕቲ 2500 ግራም እንተኮይኑ ዋና ተፀናዕቲ ኣብ ዝብል ሳፅን ምልክት ኣቅምጥ/ጢ ፤ ዕሸል እንትዉለ ክብደቱ ልዕሊ 2500 ግራም እንተኮይኑ መንፃፅሪ ተፀናዕቲ ኣብ ለሳፅን ምሌክት ኣቅምጥ/ጢ  1. ዋና ተፅናዕቲ (ኬዝ)  2. መነፃፅሪ ተፅናዕቲ (ኮንትሮል)  ትካሌ ጥዕና /ዒይነት ትካል/ ኮድ  ____________/__________/_________ | | | |
| --- | --- | --- | --- |
| 102 | ፆታ ናይቲ ህፃን እንታይ እዩ? | 1. ተባ  2. ኣነ |  |
| 103 | ሕንጦ ኣብ ክንደይ ሶሙንተወሊዱ(ዕድመ ጥንሲ)? ኣዶ ካብ ዘተርኣይ ናይ መወዲእታ ወርሓዊ  ፅግይ ተሊዒካ ብምቁፃር ወይ ካብ  መዝግብ ዉፅኢት ኣልትራሳዉንድ  ብምዉሰድ ገብጥ። | ዕድመ ጥንሲ ብሰሙናት: ________ |  |
| ክፍሊ II መረዲእታ ማሕበራዊ ኩነታት ኣዶ | | | |
| ኮድ | ሕቶታትን መግለፅታትን | መማረፅቲ መልስታት | ዝለል |
|  |  |  |  |
| 201 | ዕድመ ኣዶ | ዕድመ : _______ |  |
| 202 | ሃይማኖትኪ እንታይ እዩ? | 1. ኦርቶድክስ 2. እስሌምና  3. ካቶሊክ 4. ፕሮቴስታንት  5. ካሌእ |  |
| 203 | ናይ ኣይናይ ብሄረሰብ ኣባል ኢኪ? | 1. ትግራይ 2. ኣምሓራ   3. ዓፋር 4. ኦሮሞ  5. ካሌእ |  |
| 204 | ትነብርሉ ኣበይ እዩ? | 1. ከተማ 2. ገጠር |  |
| 205 | ኩነታት ሓዲርኪ እንታይ እዩ? | 1. ባዓልቲ ሓዳር 2. ዘይተመርዐወት  3.ዝተፋተሐት 4. በዓል ገዝአን ዝሞተን |  |
| 206 | ኩነታት ትምህርትኪ እንታይ እዩ? | 1. ምፅሓፍ ምንባብ ዘይትክእል  2.ቀዳማይብርኪት/ዘጠናቀቀት  3. ምፅሓፍ ምንባብ ትክእል  4. ካሌኣይ ብርኪ ት/ቲ ዘጠናቀቀት  5. ኮሌጅ/ዩንቨርስቲ ዘጠናቀቀት |  |

| 207 | ስራሕኺእንታይእዩ? | 1 ስራሕየብለይን 2 ነጋዴ  3 መንግስቲ ሰራሕተኛ  4 ካሊእግለፅ____________ |  |
| --- | --- | --- | --- |

| ክፍሊIII ኩነታት ሕርስን ጥንስን | | |  |
| --- | --- | --- | --- |
| ኮድ | ሕቶታትን መግለፅታትን | መማረፅቲ መልስታት | ዝለል |
| 301 | ቅድሚ ሐዚ ክንደይ ወሊድኪ | 1.1 2.2-3  3.4-5 4.>=6 |  |
| 302 | እዚን ቕድሚኡ ዘሎ ቆልዓ ኣብ ክንደይ ወርሒ ኣፈላላየይ ወሊድኪዮም? | ­____________ | ናይ መጀመርያ ቆልዓ እንተኮይኑ ናብ 303ዝለል |
| 303 | ምግንጸል ጥንሲ አጋጢሙኪ ይፈልጥ ዶ ? | 1:እወ  2: ኣይፋሉን | አይፋሉን  እንተኾይኑ ናብ305ዝለል |
| 304 | መልስኪ እወ እንተኮይኑ ክንደይ ጊዜ | በዝሒ ምግንፃል ጥንሲ----------- |  |
| 305 | ክትትል ጥንሲ ነይሩኪ ዶ? | 1:እወ 2: ኣይፋሉን | አይፋሉን  እንተኾይኑ ናብ307ዝለል |
| 306 | መልስኪእወእንተኮይኑክንደይጊዜ | በዝሒክትትልጥንሲ----------- |  |
| 307 | ኣብ ናይ ሐዚ ጥንስኺ ምስ ጥንሲ ተተሓሒዙ ዘጋጠመኪ ናይ ጥዕና ፀገም ኣሎ ዶ? | 1:እወ 2: ኣይፋሉን | አይፋሉን  እንተኾይኑ ናብ308ዝለል |
| 308 | መልስኪእወእንተኮይኑእንታይዓይነትሕማምኣጋጢሙኪ | 1 ,ቅድሚ ሕርሲ ደም ምፍሳስ  2 ,ቅድሚ ሕርሲቀስታንስቶ ምፍሳስ  3,ፀቕጢ ደም  4,ሕማም ስከር  5,ካሊእ ግለፅ |  |
| 309 | ሕዱር ሕማማት ኣለሰኪ ተባሂልኪ ትፈለጢ ዶ? | 1:እወ 2: ኣይፋሉን | አይፋሉን  እንተኾይኑ ናብ 310ዝለል |
| 311 | መልስኪ እወ እንተኮይኑ እንታይ ዓይነትሕዱር ሕማም | 1ፀቕጢ ደም 2 ሕማም ስከር  3 ደም ምንኣስ  4ናይ ሳምባ ሕማም  5 ካሊእ ግለፅ |  |
|  | **ክፍሊ IV *ናይ ኣዶ ኩነታት ልምዲ*** | | |
| ኮድ | ሕቶታትን መግለፅታትን | መማረፅቲ መልስታት | ዝለል |
| 401 | ኣብ እዋን ጥንሲ ኣልኮል ዘለዎ መስተ ትሰተይ ዶነይርኪ? | 1. እወ 2. ኣይፋሉን | አይፋሉን  እንተኾይኑ ናብ403ዝለል |
| 402 | መልስኪ እወ እንተኮይኑ ክንደይ ጊዜ ክትወስዲ ነይርኪ | 1.ሓደ ግዘ 2በቢወረሒ  3በቢሰሙን 4 በቢመዓልቲ |  |
| 403 | ኣብ እዋን ጥንሲ ሲጋራ ተትክኺ ዶ ነይርኺ?? | 1. እወ  2. ኣይፋሉን | አይፋሉን  እንተኾይኑ ናብ501ዝለል |
| 404 | መልስኪ እወ እንተኮይኑ ክንደይ ጊዜ ዝኣክል ክትወስዲ ነይርኪ | 1.ሓደ ግዘ 2 በቢወረሒ  3 በቢሰሙን 4 በቢመዓልቲ |  |
| **ክፍሊ V ዓቀን *ኩነታት*  ኣመጋግባ ኣዶ** | | | |
| ኮድ | ሕቶታትን መግለፅታትን | መማረፅቲ መልስታት | ዝለል |
| 501 | ኣብ ጊዜ ፅንስኪ ንደም ምንኣሰ ተባሂሉ ኣይረን ፎስፌት ታብሌት ወሲድኪዶ ኔይርኪ | 1:እወ 2: ኣይፋሉን | አይፋሉን  እንተኾይኑ ናብ 503 ዝለል |
| 502 | መልስኪ እወ እንተኮይኑ ክንደይ ታብሌት ወሲድኪ | 1. <60 ታብሌት  2. 60-90 ታብሌት  3. >90 ታብሌት |  |
| 503 | ኣብ ናይ መደመረያ ክትትል ፅንስኪ ክብደትኪ ተመዚንኪ ዶ ነይርኪ? | 1:እወ 2: ኣይፋሉን | አይፋሉን  ናብ505 ዘለል |
| 504 | መልስኪ እወ እንተኮይኑ ክብደትኪ ክንደይ ነይሩ | ---------------- |  |
| 505 | ኣብ ናይ መጨረሻ ክትትል ፅንስኪ ክብደትኪ ተመዚንኪ ዶ ነይርኪ? | 1:እወ 2: ኣይፋሉን | አይፋሉን  እንተኾይኑ ናብ507 ዘለል |
| 506 | መልስኪ እወ እንተኮይኑ ክብደትኪ ክንደይ ነይሩ | ---------------- |  |
| 507 | ቁመት | ----------- |  |
| 508 | መጠንወሰኽክብደትድሕሪፅንሲ | ______________ |  |
